# Combinatorial control of gene expression in *Aspergillus niger* grown on sugar beet pectin

**DOI:** 10.1038/s41598-017-12362-y

**Published:** 2017-09-27

**Authors:** Joanna E. Kowalczyk, Ronnie J. M. Lubbers, Mao Peng, Evy Battaglia, Jaap Visser, Ronald P. de Vries

**Affiliations:** Fungal Physiology, Westerdijk Fungal Biodiversity Institute & Fungal Molecular Physiology, Utrecht University, Utrecht, The Netherlands

## Abstract

*Aspergillus niger* produces an arsenal of extracellular enzymes that allow synergistic degradation of plant biomass found in its environment. Pectin is a heteropolymer abundantly present in the primary cell wall of plants. The complex structure of pectin requires multiple enzymes to act together. Production of pectinolytic enzymes in *A*. *niger* is highly regulated, which allows flexible and efficient capture of nutrients. So far, three transcriptional activators have been linked to regulation of pectin degradation in *A*. *niger*. The L-rhamnose-responsive regulator RhaR controls the production of enzymes that degrade rhamnogalacturonan-I. The L-arabinose-responsive regulator AraR controls the production of enzymes that decompose the arabinan and arabinogalactan side chains of rhamnogalacturonan-II. The D-galacturonic acid-responsive regulator GaaR controls the production of enzymes that act on the polygalacturonic acid backbone of pectin. This project aims to better understand how RhaR, AraR and GaaR co-regulate pectin degradation. For that reason, we constructed single, double and triple disruptant strains of these regulators and analyzed their growth phenotype and pectinolytic gene expression in *A*. *niger* grown on sugar beet pectin.

## Introduction

Filamentous fungi secrete an arsenal of enzymes that decompose plant polymers such as storage (starch, inulin) and cell wall polysaccharides (cellulose, hemicellulose, pectin). These enzymes have been commercially used in many industrial applications, from food-processing (e.g. baking, cheese making) to biotechnology (e.g. conversion of lignocellulose to biofuel)^1^. Pectins are the most complex polysaccharides found in plant cell walls. They are composed of four structural elements: homogalacturonan (HGA), xylogalacturonan (XGA), rhamnogalacturonan I (RG-I) and rhmnogalacturonan II (RG-II)^2^. HG is a linear polymer of α-1,4-linked D-galacturonic acid units, which can be partially methyl esterified and acetylated. XGA is HG substituted with β-1,3-linked D-xylose units. RG-I contains a backbone of α-1,4-linked D-galacturonic acid and α-1,2-linked L-rhamnose units with large arabinan and arabinogalactan side chains attached to L-rhamnose residues. The backbone of RG-II consists of α-1,4-linked D-galacturonic acid units with side chains composed of at least 12 different sugars, including rare ones such as aceric acid, apiose and 3-deoxy-manno-octulosonic acid (KDO)^2,3^. The content and structure of pectin differ depending on plant species, tissue and even growth stage. Cell walls of some fruits (such as apple and citrus) and vegetables (sugar beet) are especially rich in pectin^2^. Despite the rather intricate composition, pectins are easily decomposed by several *Aspergillus* species^4,5^ (http://www.fung-growth.org). A genomic survey revealed that *Aspergillus niger* has over 60 genes encoding characterized and putative enzymes related to pectin degradation^6,7^. For comparison, only 18, 26 and 19 genes have been found in the genomes of *Podospora anserina*, *Trichoderma virens* and *Neurospora crassa* ^5,6,8^, respectively. Because of this extraordinary pectin degradation potential, pectinases from *A*. *niger* are commonly used in extraction, clarification and modification of fruit juices^9^.

*A*. *niger* regulates production of plant polysaccharide degrading enzymes at the transcriptional level to ensure that the right proteins are produced at the right time. Transcription Factors (TFs) are regulatory proteins that bind to conserved motifs upstream from the ATG and activate or repress gene expression. Several sugar-specific TFs have been identified in *A*. *niger* ^10^. These TFs themselves are activated by the presence and concentration of monomeric sugars or products thereof formed intracellularly. Those signal molecules might be released from complex polysaccharides by the action of enzymes, constitutively present at low levels. Some endo-polygalacturonases, such as PgaA, PgaB, are believed to “scout” the environment and release D-galacturonic acid^11^, while other studies suggested that the inducers are released by enzymes appearing due to a starvation response^12,13^. Large polysaccharides, such as pectin, have potential to release multiple inducers simultaneously. This will result in a complex transcriptional response including activation, repression and de-repression mechanisms mediated by several TFs.

So far, three transcriptional activators (GaaR, RhaR and AraR) and two transcriptional repressors (GaaX, CreA) have been linked to pectin degradation in *A*. *niger* ^14–18^. The main component of pectin, D-galacturonic acid, specifically up-regulates the expression of a subset of pectinolytic genes^17,19^. Further studies revealed that expression of those pectinases is controlled by GaaR and induced by 2-keto-3-deoxy-L-galactonate, an intermediate of the D-galacturonic acid catabolic pathway^14,20^. The majority of GaaR-dependent genes encode enzymes needed for decomposition of HG, such as exo-polygalacturonases (*pgaX*, *pgxA*, *pgxB*, *pgxC*), endo-polygalacturonases (*pgaI*, *pgaC*, *pgaE*), pectin methyl esterases (*pmeA*, *pmeB*, *pmeC*) and pectin lyases (*pelA*, *pelD*, *pelF*)^14^. Additionally, GaaR regulates expression of genes (putatively) involved in D-galacturonic acid uptake (*gatA*, NRRL3_08663, NRRL3_04281) and metabolism (*gaaA*, *gaaB*, *gaaC*, *gaaD/larA*)^14,21^. A conserved GARE motif CC[ACTG]CCAA was found in the promoters of all genes up-regulated in the presence of D-galacturonic acid^21^ and this regulatory element was shown to be essential for GaaR-dependent gene expression in *A*. *niger* ^18^.

Release, uptake and metabolism of another building block of pectin, L-rhamnose, is regulated by RhaR^15,22^. Gruben *et al*.^15^ showed that RhaR activates expression of genes encoding enzymes acting mainly on RG-I, such as exo-rhamnogalacturonases (*rgxA*, *rgxB*, *rgxC*), rhamnogalacturonan lyase (*rglB*), rhamnogalacturonan acetyl esterase (*rgaeB*), unsaturated rhamnogalacturonan hydrolase (*urhgA*) and α-rhamnosidases (NRRL3_02162, NRRL3_07520, NRRL3_04245, NRRL3_06304, NRRL3_03279). The third TF involved in pectin degradation, AraR, activates expression of genes encoding enzymes needed for L-arabinose release from RG-I side chains (*abfA*, *abfB*) and its metabolism (*larA/gaaD*, *ladA*, *lxrA*, *xdhA*, *xkiA*)^16^. While the AraR regulatory motif is currently not known, six conserved motifs were found in the promoters of AraR-regulated metabolic genes *in silico* ^23^. A recent study reported that expression of most pectinases is also affected by the D-galacturonic acid responsive repressor, GaaX, which was shown to be under control of GaaR in *A*. *niger* ^24^. The activator-repressor model proposed by Niu el al. (2017) states that expression of those genes is an effect of inducer binding to the GaaX repressor which is released from GaaR resulting in GaaR to become active. Several D-galacturonic acid-induced pectinolytic genes (*pgaI*, *pgaII*, *pgaC*, *pgaE*, *pelB*, *pelC*, *plyA*, *rhgA*, *rgaeA*) were also shown to be under control of the general carbon catabolite repressor, CreA^17,18^.

Regulation of eukaryotic gene expression often involves several TFs, resulting in a combined effect^25^. Understanding the cooperative action of sugar-specific TFs in the regulatory network and how they affect gene expression in filamentous fungi has been a challenge and so far only a few studies have addressed this topic. In *A*. *nidulans*, AraR and XlnR were shown to co-regulate metabolic genes involved in pentose catabolism, while GalR, AraR and XlnR co-regulate D-galactose conversion^26,27^. Another study showed that an interaction of two TFs, ClrB and McmA, is necessary for regulation of the endoglucanase encoding genes *eglA* and *eglB* in *A*. *nidulans* ^28^, while in *A*. *niger*, expression of *cbhA*, *eglC* and *xynA* was shown to be co-dependent on both XlnR and ClrB^29^.

The aim of this study was to better understand the contribution of the transcriptional activators GaaR, RhaR and AraR in regulating pectin degradation in *A*. *niger*. For this, single, double and triple mutants of g*aaR*, *rhaR* and *araR* were constructed and their phenotype and transcriptional response on pectin was analyzed. Moreover, this study provides more insight into combinatorial control of pectinolytic gene expression and suggests genes regulated by two and/or three TFs.

## Results and Discussion

### All *A*. *niger* strains in which *gaaR* is deleted have reduced growth on pectin

**Figure 1:**
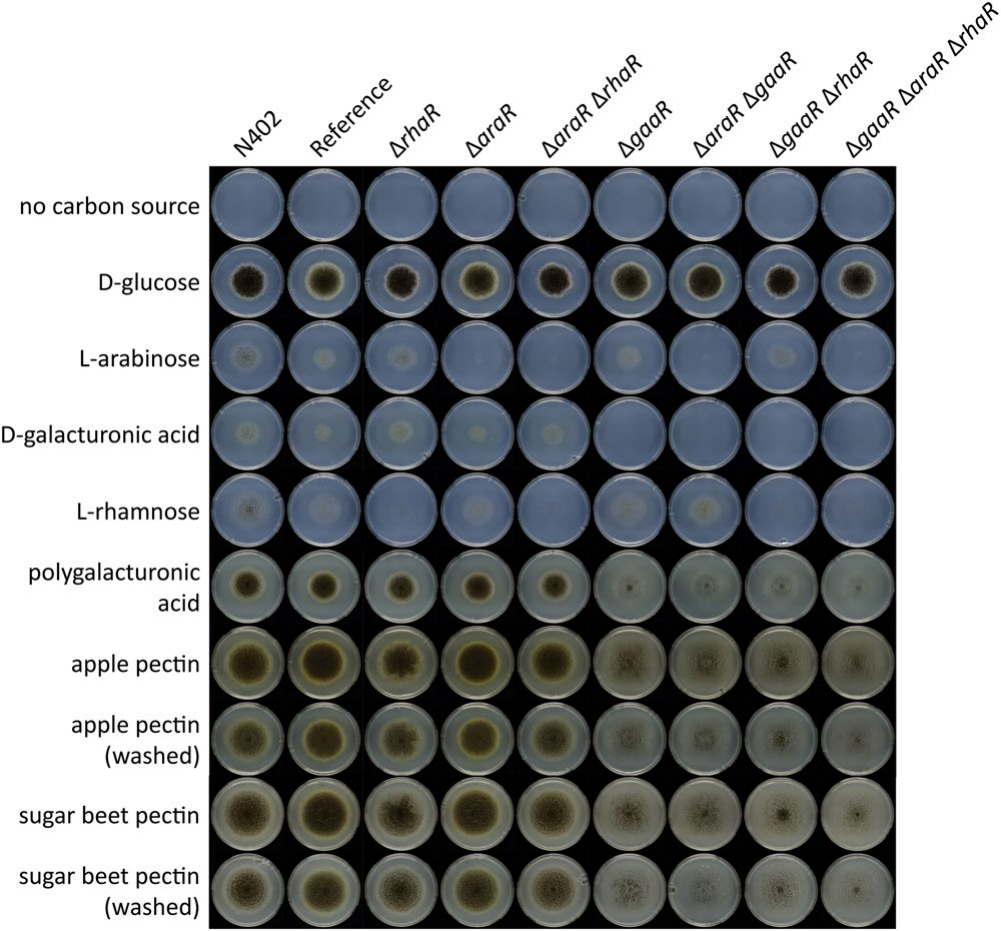
Phenotypic analysis of *A*. *niger* reference strain and regulatory mutants on pectin and pectin-related carbon sources.

To study the relative contribution of GaaR, AraR and RhaR in regulation of pectin degradation, single, double and triple deletion strains of those TFs (listed in Table 1) were constructed and verified by Southern blotting. The phenotype of the reference strain and regulatory mutants was then compared on monomeric and polymeric carbon sources (Fig. 1).

**Table 1:** *A*. *niger* strains used in this study.

Strain ID	CBS accession number	Genotype	Reference
N402	141247	*cspA1*	53
N593 Δ*kusA* (Reference strain)	138852	*cspA1*, *pyrG* ^−^, *kusA::amdS*	54
Δ*gaaR*	141258	*cspA1*, *pyrG* ^−^, *kusA::amdS*, *ΔgaaR*::*pyrG*	14
Δ*araR*	142671	*cspA1*, *pyrG* ^−^, *kusA::amdS*, *ΔaraR*::*hph*	This study
Δ*rhaR*	137440	*cspA1*, *pyrG* ^−^, *kusA::amdS*, *ΔrhaR::pyrG*	15
Δ*araR*Δ*gaaR*	142672	*cspA1*, *pyrG* ^−^, *kusA::amdS*, *ΔgaaR::pyrG*, *ΔaraR*::*hph*	This study
Δ*araR*Δ*rhaR*	142673	*cspA1*, *pyrG* ^−^, *kusA::amdS*, *ΔrhaR::pyrG*, *ΔaraR*::*hph*	This study
Δ*gaaR*Δ*rhaR*	142674	*cspA1*, *pyrG* ^−^, *kusA::amdS*, *ΔrhaR::pyrG*, *ΔgaaR::hph*	This study
Δ*gaaR*Δ*araR*Δ*rhaR*	142675	*cspA1*, *pyrG* ^−^, *kusA::amdS*, *ΔrhaR::pyrG*, *ΔaraR*::*hph*, *ΔgaaR::phle*	This study

The growth on D-glucose was used as an internal control and was similar in all the strains. Growth was abolished in the triple mutant, Δ*gaaR*Δ*araR*Δ*rhaR*, on D-galacturonic acid, L-arabinose and L-rhamnose because conversion of those monosaccharides relies on GaaR, AraR and RhaR, respectively^14–16^. Reduction in growth and sporulation on polygalacturonic acid (PGA), apple pectin (AP) and sugar beet pectin (SBP) was observed in all strains where *gaaR* was deleted (Fig. 1), most strongly in the triple mutant, Δ*gaaR*Δ*araR*Δ*rhaR*. This might be explained by (1) co-dependency of pectinolytic gene expression on GaaR, AraR and RhaR, or (2) abolished metabolism of D-galacturonic acid, L-arabinose and L-rhamnose, which contributes to reduced growth. Reduction in sporulation on pectin was more pronounced in the Δ*araR*Δ*gaaR* and Δ*gaaR*Δ*rhaR* double mutants than in the Δ*gaaR* single mutant. Among the double mutants, the phenotype of the Δ*araR*Δ*gaaR* was altered more strongly, suggesting that AraR has more influence in regulating pectin degradation than RhaR. The growth of Δ*araR*Δ*rhaR* on PGA, AP and SBP was similar to the reference strain confirming that GaaR is the main TF involved in pectin degradation.

### The triple mutant has a reduced ability to hydrolyze pectin

To evaluate the hydrolytic activity of the *A*. *niger* reference strain and regulatory mutants against pectin, the culture filtrates of those strains grown on SBP for 8 h were used for saccharification and the enzyme products were identified with HPLC. Analysis of carbohydrate monomers released from SBP by the filtrate of the reference strain showed that D-galacturonic acid, L-arabinose and D-galactose, but not L-rhamnose, were produced.

**Figure 2:**
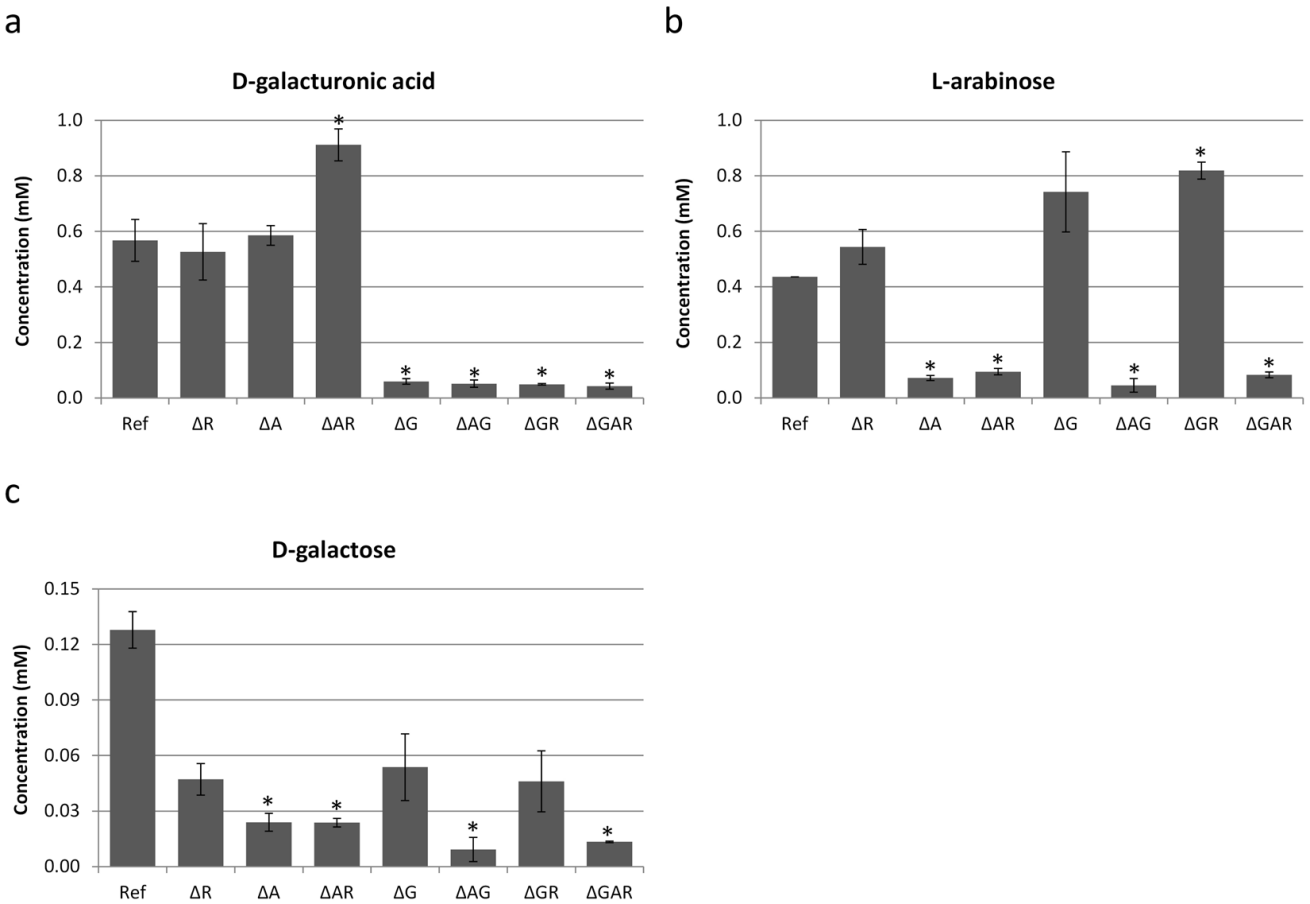
Monomeric sugars released from 3% SBP with crude extracellular enzyme mix from 8 h cultures of *A*. *niger* reference strain and regulatory mutants. Significant changes (*P*-values ≤ 0.05) between the mutant and the reference strain were indicated by an asterisk.

The hydrolytic activities necessary for D-galacturonic acid release from SBP were reduced in all Δ*gaaR* mutants (Fig. 2A), which can be explained by the fact that GaaR controls the expression of most genes encoding enzymes that act on HG^14^. An increased amount of D-galacturonic acid was detected in the culture filtrate of Δ*araR*Δ*rhaR*, but not in the single Δ*araR* or Δ*rhaR* mutants. Transcriptome data showed that several exo- and endo-galacturonases are up-regulated in these strains, and the strongest effect was observed in the double mutant (see below). The residual amount of D-galacturonic acid released in the case of the triple mutant might be caused by the action of constitutively expressed pectinases, which are essential for release of the first signal molecules.

The hydrolytic activities resulting in L-arabinose release from pectin was reduced in all strains in which *araR* was deleted (Fig. 2B). Interestingly, the Δ*gaaR*Δ*rhaR* culture filtrate released more L-arabinose than that of the reference strain. This correlates with increased expression of genes encoding L-arabinose releasing enzymes (*abfB*, *abnA*, *abnB*) (see below). The residual amount of L-arabinose in the case of the triple mutant might be released by (1) action of arabinanolytic enzymes regulated by other TFs, e.g. XlnR^30^, (2) action of arabinanolytic enzymes which are constitutively expressed.

The culture filtrates of the GaaR, AraR and RhaR regulatory mutants were able to release D-galactose from SBP (Fig. 2C). However, the amount of D-galactose was considerably reduced in the case of all Δ*araR* mutants compared to the reference strain (Fig. 2C). This might be linked to the reduction of expression of three β-galactosidases (*lacA*, *lacB*, *lacC*) in most strains in which AraR was deleted (see below).

We were not able to detect any free L-rhamnose, which indicates that enzymatic activities needed to release it from SBP lack in the culture filtrates harvested after 8 h of growth. This correlates with the transcriptome data, which showed that the majority of genes encoding rhamnose-releasing enzymes were lowly expressed (FPKM 1–20) in the reference strain grown on SBP for 2 and 8 h (see below). Taking into account that L-rhamnose is located in the backbone of heavily branched RG-I, appearance of these activities may require prolonged cultivation time of the strains on SBP before harvesting.

### GaaR, AraR and RhaR all contribute to regulation of pectinolytic genes

To gain insight into the regulation of pectin-degrading enzymes, the transcriptome response during growth on SBP was analyzed in the single, double and triple mutants of GaaR, AraR and RhaR. We focused on previously described^19^ and putative (www.cazy.org) genes encoding enzymes involved in pectin degradation and metabolism of released monomers, because their expression is directly linked to survival of the mutants on SBP. This approach allowed identification of genes co-dependent on two and three TFs. A cut-off of fold change > 1.5 between the FPKM values of the mutants and the reference strain and a *P*-value ≤ 0.05 were used to identify differentially expressed genes. Expression of *gaaR*, *araR* and *rhaR* was abolished in all corresponding mutants confirming that the correct gene deletions were made (Supplementary dataset S1). Interestingly, expression of *gaaR* was 2-fold reduced (*P*-value 4E-05) in the Δ*araR* strain after 2 h of growth on SBP, which resulted in an indirect effect on the expression of GaaR-dependent genes.

**Figure 3:**
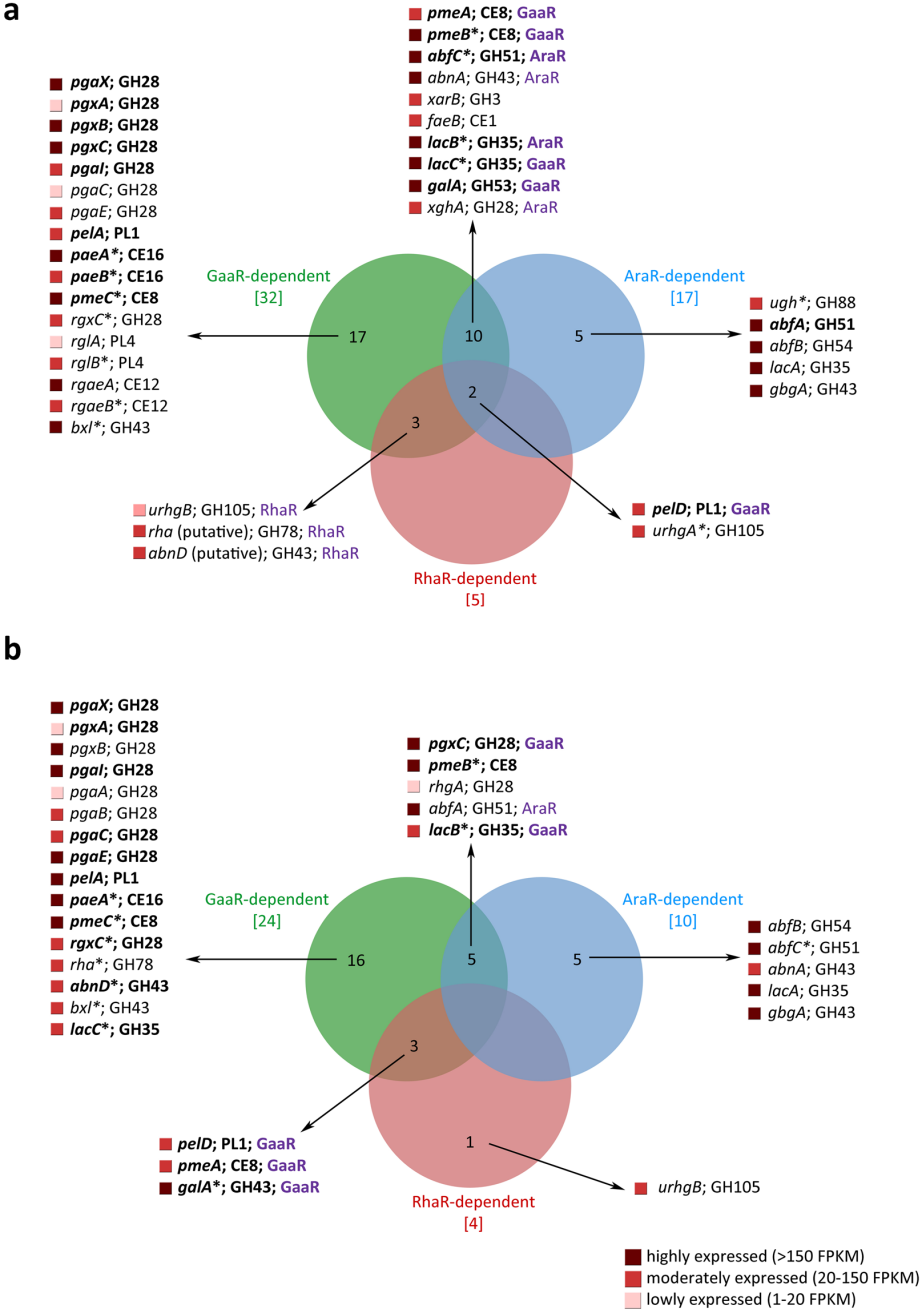
Venn diagram showing an overlap between GaaR-, AraR- and RhaR-dependent genes in *A*. *niger* grown on SBP for 2 h (**a**) and 8 h (**b**). Genes with no biochemical validation available are marked by an asterisk. The gene abbreviations and CAZy family numbers are listed in Supplementary datasets S2 and S3. Genes that were previously identified as GaaR-dependent on SBP by Alazi *et al*.^14^ were marked in bold. Expression of genes under combinatorial control of two or three TFs was evaluated and, if possible, the predominant activator was indicated (violet letters behind the gene name).

**Figure 4:**
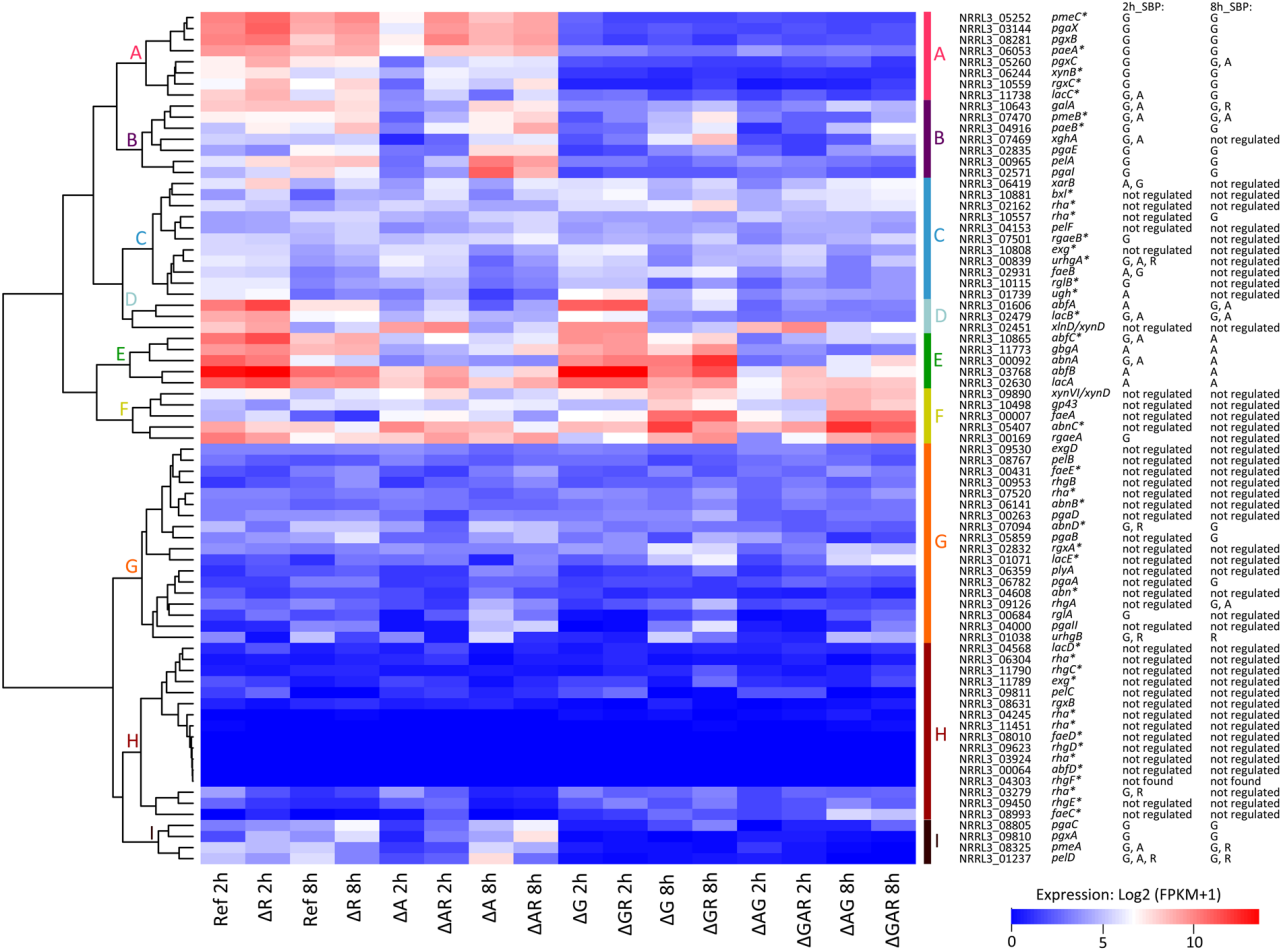
Hierarchical clustering of genes linked to pectin utilization in the reference strain and regulatory mutants grown on SBP for 2 and 8 h. The color code displayed represents averaged and logged expression values (FPKM + 1) of duplicates. Genes classified as GaaR- (G), AraR- (A), RhaR-dependent (R) or not regulated after 2 and 8 h on SBP are indicated. Genes with no biochemical validation available are marked by an asterisk. The (putative) function of the genes can be found in Supplementary dataset S12.

The influence of *gaaR*, *araR* and *rhaR* deletions on expression of pectinolytic genes was analyzed over time (2 and 8 h). After 2 h on SBP, expression of 37 genes (49%) was regulated by at least one of the TFs tested, 29 genes (38%) were not regulated by any of the TFs and 10 genes (13%) were considered not expressed (FPKM < 1) (Supplementary dataset S2). The majority of the genes encoding HG-active enzymes were down-regulated in all strains where *gaaR* was deleted confirming that GaaR is the main TF regulating D-galacturonic acid release from pectin (Figs 3A and 4). One gene (NRRL3_01739), encoding a putative unsaturated glucuronyl hydrolase, was down-regulated in all *araR* deletion strains (Fig. 4 Cluster C). Interestingly, expression of two pectin methyl esterases (*pmeA*, *pmeB*) was reduced in all strains where GaaR and/or AraR was absent while one pectin lyase (*pelD*) was down-regulated in all mutants. These genes are likely to be under combinatorial control of the TFs on SBP. The change in expression of genes dependent on two or three TFs was analyzed and, if possible, the TF with the strongest effect was determined. GaaR was the predominant activator of *pmeA*, *pmeB* and *pelD* expression after 2 h of growth on SBP (Fig. 3A).

Among genes encoding RG-I-active enzymes, five (*rgxC*, *rglA*, *rglB*, *rgaeA*, *rgaeB*) were down-regulated in all strains in which *gaaR* was deleted (Fig. 4 Clusters A, C, F and G). These genes (except *rglA*) were previously shown to be RhaR-dependent when grown on L-rhamnose. This might be caused by differences in gene regulation when monomeric and complex sugars are used, as shown previously for AraR and XlnR^30^. Another explanation might be the lack of detectable free L-rhamnose in our cultures (Fig. 2), which likely decreased the overall effect of RhaR on gene expression. Expression of two genes (*urhgB*, NRRL3_03279) was reduced when either *gaaR* and/or *rhaR* were absent, with RhaR being the predominant TF of the pair. Interestingly, expression of the *urghA* gene was reduced in Δ*araR*Δ*gaaR* and/or Δ*gaaR*Δ*rhaR* double mutants but not in the single *araR*, *gaaR* or *rhaR* deletion strains.

Among genes encoding enzymes active on pectin side-chains, four (*abfA*, *abfB*, *lacA*, *gbgA*) showed reduced expression in all strains in which *araR* was absent (Fig. 4 Clusters E and D), while seven (*abfC*, *abnA*, *xarB*, *faeB*, *lacB*, *lacC*, *galA*) were down-regulated when AraR and/or GaaR were deleted (Fig. 4 Clusters A, B, C, D and E). The expression of *abnD* was reduced when *gaaR* and/or *rhaR* were deleted (Fig. 4 Cluster G) and a putative β-xylosidase encoding gene (*xynB*, NRRL3_06244) was down-regulated in all strains where *gaaR* was absent (Fig. 4, Cluster A). Expression of the xylogalacturonase-encoding gene (*xghA*), was reduced in all strains in which *gaaR* and/or *araR* was absent (Fig. 4 Cluster B).

After 2 h of growth on SBP, the majority of pectinolytic genes that are not regulated by any of the TFs tested were lowly expressed (FPKM 1–20), including four endopolygacturonases (*pgaII*, *pgaA*, *pgaB*, *pgaD*), three pectin lyases (*pelB*, *pelC*, *pelF*), one pectate lyase (*plyA*), one exo-rhamnogalacturonase (*rgxA*), three endo-rhamnogalacturonases (*rhgA*, *rhgB*, *rhgE*), two endoarabinanases (*abnB*, NRRL3_04608) and two β-galactosidases (*lacD*, *lacE*) (Fig. 4 Clusters G, C and H; Supplementary dataset S2). The enzymatic activities encoded by these lowly expressed genes will play an important role in releasing the initial monomeric sugars (inducers) from pectin as described previously for PgaA and PgaB^11^.

After 8 h of growth on SBP, expression of 30 pectinolytic genes (39%) was regulated by at least one of the tested TFs, 34 genes (44%) were not regulated by any of the TFs and 12 genes (16%) were considered not expressed (FPKM < 1) (Supplementary dataset S3). Expression of 11 genes encoding HG-active enzymes was reduced in all strains in which *gaaR* was deleted (Figs 3B and 4). Nine of them (*pgaX*, *pgxA*, *pgxB*, *pgaI*, *pgaC*, *pgaE*, *pelA*, *paeA*, *pmeC*) were under control of GaaR at both time points tested (2 and 8 h; Fig. 4 Clusters A, B and I). The other two endo-polygalacturonase encoding genes (*pgaA*, *pgaB*) were only GaaR-regulated after 8 h of growth on SBP (Fig. 4 Cluster G). Expression of *pgxC*, regulated by GaaR after 2 h, was after 8 h reduced in all strains in which *gaaR* and/or *araR* were deleted, but GaaR remained the predominant TF (Fig. 4 Cluster I). Interestingly, after 2 and 8 h on SBP the expression of *pmeB* was only reduced when both *gaaR* and *araR* were deleted (Fig. 4 Cluster B). Two genes (*pelD*, *pmeA*) were down-regulated when both *gaaR* and *rhaR* were deleted, and in both cases GaaR had the predominant effect (Fig. 4 Cluster I). After 8 h of growth on SBP, only four genes encoding RHG I-active enzymes were affected by GaaR, AraR or RhaR. Two genes (*rgxC*, NRRL3_10557) were down-regulated in all *gaaR* deletion strains, while one (*urhgB*) was affected by the *rhaR* deletion and one (*rhgA*) was affected when both *gaaR* and *araR* were deleted (Fig. 3B).

In summary, we identified the set of pectinolytic genes regulated by GaaR, AraR and RhaR in *A*. *niger* grown on SBP. We also identified genes that are under combinatorial control of two or three TFs. Moreover, we showed that the transcriptional regulation of pectinolytic genes changes over time as subsets of genes were regulated by different TFs after 2 and 8 h of growth on SBP. Our results show that all TFs contribute to pectin degradation.

### The triple mutant cannot convert the main components of pectin (D-galacturonic acid, L-arabinose and L-rhamnose)

SBP contains 55 mol % D-galacturonic acid, 17 mol % L-arabinose, 16 mol % D-galactose and 10 mol % L-rhamnose^14^. To further catabolize these monomeric sugars, *A*. *niger* must activate relevant metabolic pathways. To evaluate the metabolic potential of the strains, expression of genes involved in conversion of D-galacturonic acid (*gaaA*, *gaaB*, *gaaC*, *gaaD/larA*), L-arabinose/D-xylose (*larA/gaaD*, *ladA*, *lxrA*, *xdhA*, *xkiA*, *xyrA*), L-rhamnose (*lraA*, *lraB*, *lraC*) and D-galactose (*galK*, *galD*, *galF*, *galG*, *pgmB*, *ladB*, *xhrA*, *sdhA*) was analyzed in the regulatory mutants and the reference strain grown on SBP for 2 and 8 h.

**Figure 5:**
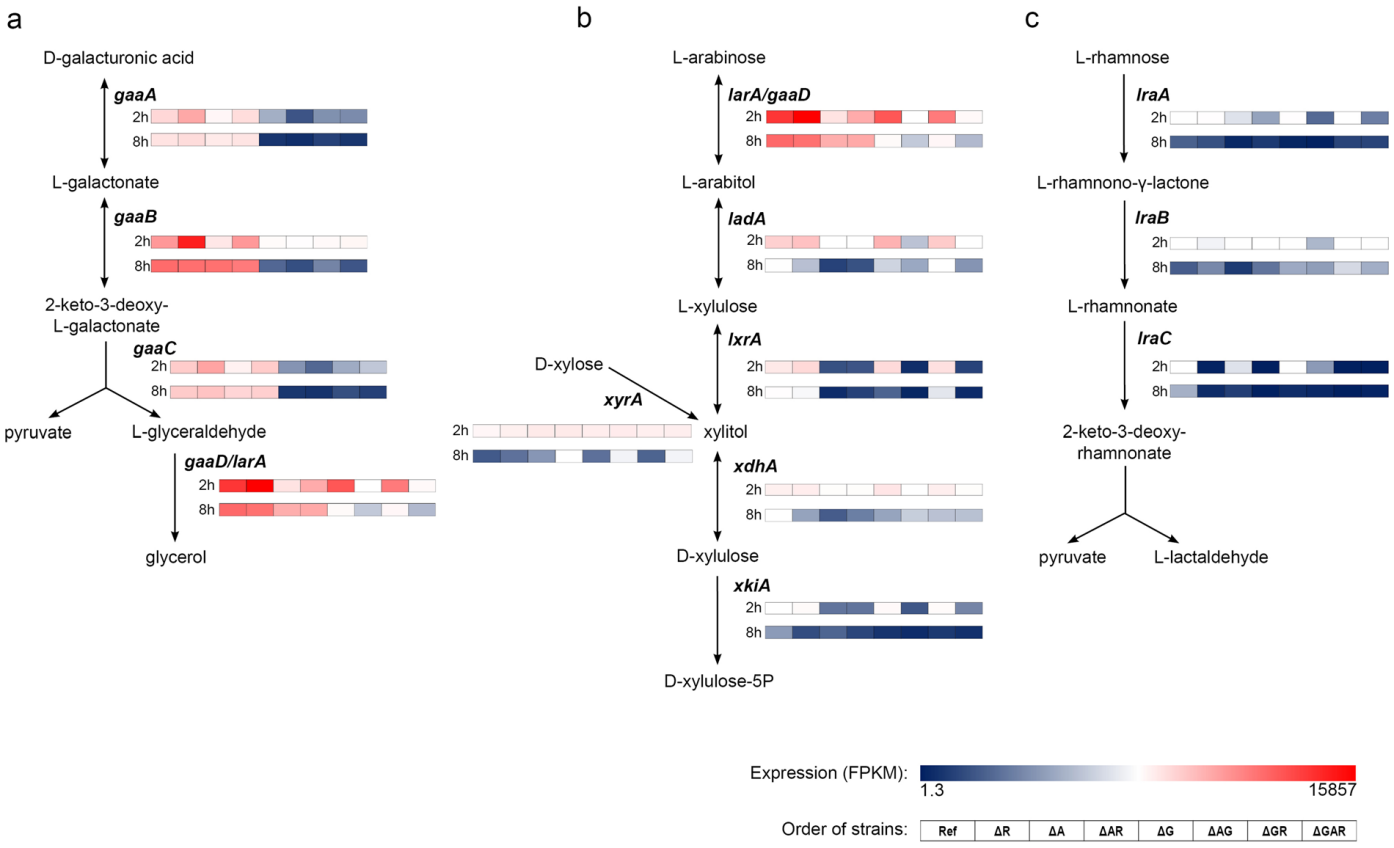
RNA-seq analysis of genes involved in D-galacturonic acid (**a**), L-arabinose/D-xylose (**b**) and L-rhamnose (**c**) conversion in *A*. *niger* reference strain and regulatory mutants grown on SBP for 2 and 8 h. Expression values (FPKM) are averages of duplicates and presented as color gradient.

Growth on D-galacturonic acid was abolished in all strains in which *gaaR* was deleted (Fig. 1), which confirms an earlier study showing that GaaR is necessary for induction of *gaaA*, *gaaB*, *gaaC* and *gaaD/larA* genes involved in D-galacturonic acid metabolism in *A*. *niger* ^14^. After 2 and 8 h of growth on SBP, expression of the first three genes involved in D-galacturonic acid metabolism (*gaaA*, *gaaB*, *gaaC*) was substantially reduced in all strains in which *gaaR* was deleted (Fig. 5, Supplementary dataset S4). As shown by Alazi *et al*.^14^, expression of *gaaD/larA* was reduced but still detectable in Δ*gaaR* grown on SBP for 2 and 8 h (Fig. 5, Supplementary dataset S4). The GaaD/LarA enzyme can convert both L-glyceraldehyde and L-arabinose and is therefore involved in the pentose catabolic pathway and the D-galacturonic acid catabolic pathway^31^. After 2 h on SBP, expression of *gaaD/larA* was reduced in Δ*araR*, Δ*araR*Δ*gaaR*, Δ*gaaR*Δ*araR*Δ*rhaR* mutants, but not in Δ*gaaR*. After 8 h on SBP, expression of this gene was affected by both *araR* and *gaaR* deletion, confirming that this gene is regulated by both AraR and GaaR. Deleting both araR and *gaaR* abolished expression of *gaaD/larA* on SBP at both time points tested.

Growth on L-rhamnose was abolished in all strains where *rhaR* was absent (Fig. 1), because RhaR controls expression of the L-rhamnose conversion pathway in *A*. *niger* ^15^. After 2 and 8 h on SBP, expression of *lraC* was abolished in all strains in which *rhaR* was deleted (Fig. 5, Supplementary dataset S4). Expression of the other two genes, *lraA* and *lraB*, was weakly affected by RhaR which indicates partial regulation when grown on complex substrates.

Growth on L-arabinose was abolished in all strains in which *araR* was absent (Fig. 1). Previous studies showed that genes involved in the pentose catabolic pathway are regulated by AraR and XlnR in *A*. *niger* grown on L-arabinose^16^. After 2 and 8 h of growth on SBP, expression of L-arabitol dehydrogenase (*ladA*) and L-xylulose reductase (*lxrA*) was very low in all strains in which *araR* was absent (Fig. 5) suggesting that these genes are under absolute control of AraR. The expression of *xyrA*, which encodes the enzyme that converts D-xylose to xylitol, was shown to be controlled by XlnR in *A*. *niger* on L-arabinose and D-xylose^16^. Accordingly, expression of this gene is not affected by deletion of GaaR, AraR or RhaR after 2 h of growth on SBP. After 8 h on SBP, expression of *xyrA* was considerably up-regulated in all strains where *araR* was absent. This might be caused by XlnR-AraR compensation effect, when in case of loss of one TF the other one takes over to maintain expression of crucial genes, as recently shown in *A*. *nidulans* ^27^. Expression of *xdhA* and *xkiA* was reduced in all Δ*araR* strains when compared to the reference strain, but the mRNA levels suggest that these genes remained active. This indicates that the *araR* deletion mutants are able to metabolize D-xylose, which is present in SBP in small amounts (0.5 mol %).

*A*. *niger* can convert D-galactose through the Leloir pathway and the oxido-reductive pathway^32^. When grown on SBP, the Leloir pathway genes were expressed at a higher level in the reference strain and the regulatory mutants suggesting that this pathway was mainly responsible for D-galactose conversion. The expression of the Leloir (*galK*, *galD*, *galF*, *galG*, *pgmB*) and the oxido-reductive (*ladB*, *xhrA*, *sdhA*) pathway genes was barely affected by *gaaR*, *araR* and *rhaR* deletions which indicates that GaaR, AraR and RhaR are not involved in regulation of D-galactose metabolism (Supplementary dataset S4). After 8 h of growth on SBP, expression of the *ladB* gene was considerably increased in all strains in which *gaaR* was absent. This corresponds with increased expression of *galX*, which encodes a TF that regulates the D-galactose oxido-reductive pathway in *A*. *niger* ^33^ (Supplementary dataset S1). Our results indicate that the Δ*gaaR*Δ*araR*Δ*rhaR* triple mutant is not able to metabolize D-galacturonic acid, L-arabinose and L-rhamnose but can still grow on pectin due to D-galactose release and metabolism.

### Expression of XlnR-dependent targets is up-regulated in *gaaR* deletion strains

**Figure 6:**
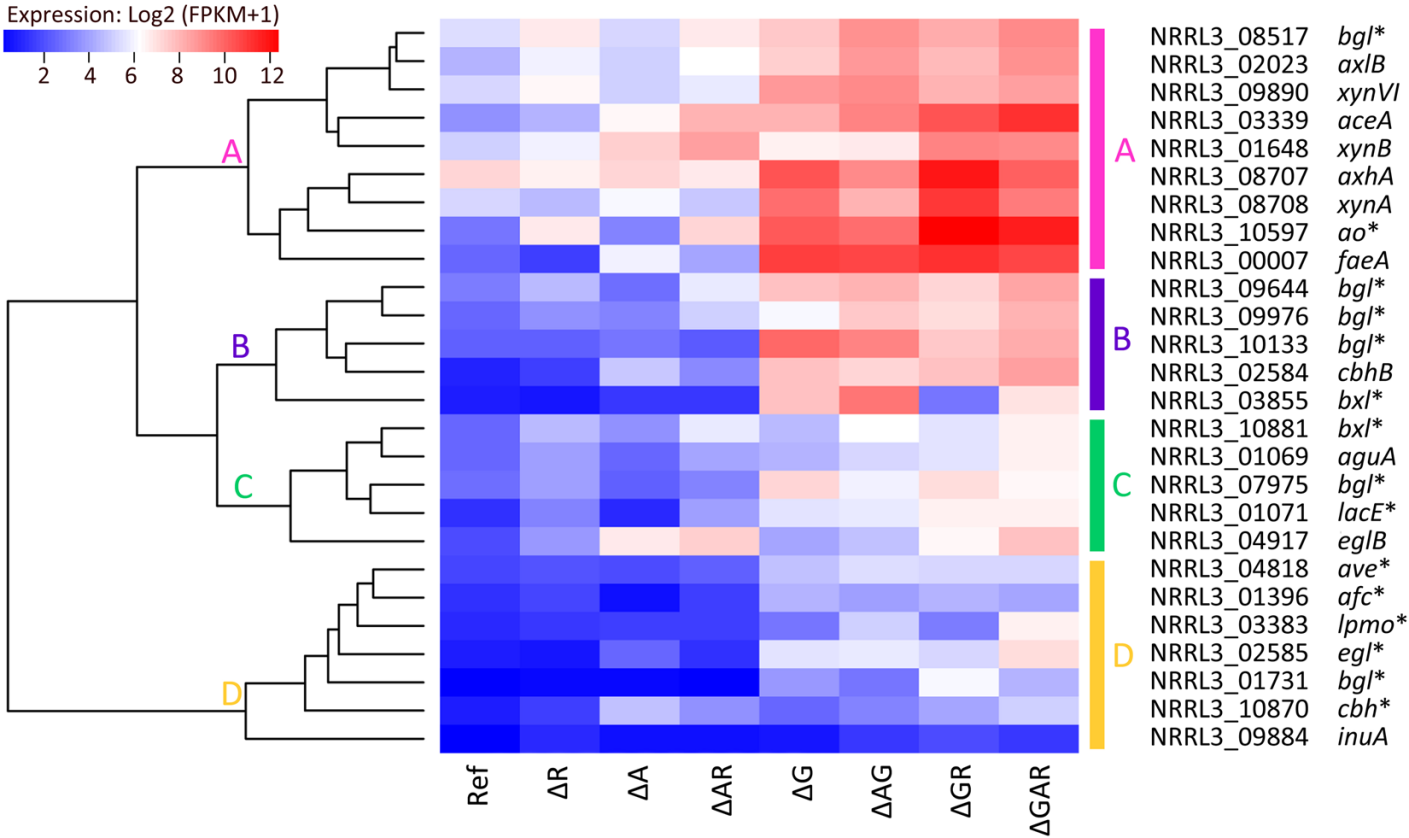
Hierarchical clustering of CAZy genes with significantly up-regulated expression levels in the triple mutant grown on SBP for 8 h. The color code displayed represents averaged and logged expression values (FPKM + 1) of duplicates. Clusters A-D can be distinguished. Genes with no biochemical validation available are marked by an asterisk. The (putative) function of the genes can be found in Supplementary dataset S12.

After 8 h of growth on SBP, expression of several genes encoding xylan and cellulose-degrading enzymes was strongly up-regulated in all strains in which *gaaR* was deleted (Fig. 6). This group included known XlnR-dependent genes, such as *faeA*, *cbhB*, *eglB*, *aguA* and *axhA* (Fig. 6 Clusters A–C)^34,35^ and correlated with increased transcript levels of the *xlnR* gene, which was 2-fold higher in the triple mutant grown on SBP for 8 h (Supplementary dataset S1). Other TF-encoding genes, *clrA* and *clrB*, involved in cellulose degradation were also up-regulated. This is most likely a consequence of the increased expression levels of *xlnR*, since XlnR was reported to regulate expression of *clrA* and *clrB* in *A*. *niger* grown on lignocellulose^29^. These results suggest that the TFs GaaR and XlnR might have an antagonistic effect in *A*. *niger*.

### Conserved regulatory motifs were found in the promoter of co-operated pectinolytic genes

Presence of a conserved regulatory element in the promoter of a gene suggests direct regulation by TF-DNA interaction^36^. While GaaR and (putative) AraR motifs have been identified before^21,23^, the RhaR consensus element remains unknown. In this study we analyzed promoters of 34 RhaR-dependent genes for *in silico* motif prediction using MEME Suite software^37^. One novel motif, TG[CAG][GTA]GGG, was identified in the promoters of 82% (28/34) of the RhaR-dependent genes with the *P*-value < 1E-04 (Supplementary dataset S5). Compared to relatively high enrichment among RhaR target genes, the motif was present in only 31% (3688/11717) promoters in the whole genome promoter search. Additionally, we investigated the evolutionary conservation of TG[CAG][GTA]GGG motif among 28 putative RhaR-dependent genes in five representative *Aspergillus* species (*A*. *niger*, *A*. *nidulans*, *A*. *aculeatus*, *A*. *fumigatus*, *A*. *oryzae*) (Supplementary dataset S5). We found the motif to be present in the majority of the orthologous genes analyzed showing high conservation among Aspergilli.

1 kb upstream regions of genes under combined control of two or three TFs were analyzed for the presence of conserved (putative) GaaR, AraR and RhaR binding motifs (overview in Table 2, Supplementary dataset S6). Four out of six putative AraR binding motifs^23^ were not found in the promoter of analyzed genes; therefore only two remaining motifs are presented in Table 2. For four genes (*pmeA*, *pmeB*, *xghA* and *abnD*) the presence of binding motifs corresponded with the expression data. The expression analysis indicated that *pmeA*, encoding pectin methyl esterase, is controlled by GaaR and AraR after 2 h and by GaaR and RhaR after 8 h of growth on SBP (Figs 3 and 4). The putative binding motifs of all three TFs were indeed identified in the *pmeA* promoter (Table 2). Similarly, the binding motifs of GaaR and AraR, but not RhaR were identified in the promoter of another pectin methyl esterase *pmeB* and this gene was shown to be co-dependent on GaaR and AraR on pectin. The expression of *xghA*, encoding xylogalacturonase, is GaaR and AraR-dependent after 2 h of growth on SBP and binding sites for both TFs were detected in the *xghA* promoter. Finally, expression of *abnD*, encoding a putative endo-1,5-alpha-arabinanase, was controlled by both GaaR and RhaR after 2 h on SBP. Again, the regulatory motifs of GaaR and RhaR were identified in this promoter. Thus, the presence of these conserved elements supported by the expression analysis strongly suggests that these genes are under combined control in *A*. *niger* grown on sugar beet pectin. For the other genes identified as co-dependent on two or three TFs on SBP, there was no correlation between the presence of the binding site and the expression. For those genes we hypothesize that (1) permitted variation in sequence of the regulatory elements occurred, (2) other, unknown regulatory elements are involved or (3) other regulatory proteins are recruited.

**Table 2:** Promoter analysis of selected pectinolytic genes. The position of the motif on the upstream strand is given with respect to the transcription start codon. The (putative) function of the genes can be found in Supplementary dataset S12.

NRRL3 gene ID	Gene name	GaaR regulatory element	AraR regulatory elements^a^		RhaR regulatory element^b^
		CC[acgt]CCAA	CCCC[atcg]CC	[ctg][tc][tca][ct][tc][cta][ct][tcg][tc][ct][tc][cta][ta][ct][ct]	TG[cag][gta]GGG
NRRL3_08325	*pmeA*	(−) 307 and (+) 982	(+) 180 and (−) 877		(+) 957
NRRL3_07470	pmeB*	(+) 388	(+) 539		
NRRL3_01237	*pelD*	(−) 409 and 465^c^		(+) 73	(−) 453 and 701
NRRL3_05260	*pgxC*	(+) 267 and (−) 642			(−) 462 and 701
NRRL3_07469	*xghA*	(−) 567	(−) 416		
NRRL3_09126	*rhgA*		(−) 363		(+) 313
NRRL3_00839	urhgA*	(−) 990		(+) 14	(+) 989 and (−) 125
NRRL3_01038	urhgB*			(+)177, 221, 218, 128 and 125	
NRRL3_03279	rha*	(+) 544	(+) 916		(+) 89 and 237 and 890
NRRL3_01606	*abfA*		(+) 133	(+) 196, 253 and 268	(+) 467 and 819
NRRL3_10865	abfC*			(−) 769, 770 and 773	(−) 195 and (+) 791
NRRL3_00092	*abnA*	(+) 999			(+) 663
NRRL3_07094	abnD*	(+) 230		(+) 488	(+) 739 and 774
NRRL3_02479	lacB*			(−) 535	(+) 13
NRRL3_11738	lacC*	(+) 266			(−) 171 and 362 and 839
NRRL3_06419	*xarB*		(+) 508 and 513 (−) 888	(+) 98, 205, 206 and 211 and (−) 998	(−) 331 and 686
NRRL3_02931	*faeB*		(−) 679	(+) 43, 47 and 48	(+) 84 and 232 and 965

^*^No biochemical validation available.

^a^Two of six putative AraR binding motif predicted *in silico* by Battaglia *et al*.^23^.

^b^Putative RhaR binding motif predicted *in silico* in this study.

^c^Not identified in our study but detected by Alazi *et al*.^14^.

In summary, we found that GaaR, AraR and RhaR all contribute to the regulation of pectin degradation in *A*. *niger* grown on sugar beet pectin. By studying single, double and triple mutants of those TFs, we identified the sets of down-regulated genes controlled by those TFs (Supplementary datasets S7 and S8). This approach leads to stronger conclusions than studying single TF knockouts as the strains are internal references for each other. Our results confirm that GaaR is the major TF involved in regulation of pectin degradation because it controls expression of the majority of genes encoding enzymes acting on HG, RG-I and the arabinan and arabinogalactan side chains of pectin. RhaR had the least impact on expression of pectinolytic genes on SBP and most of the genes encoding RG I-active enzymes were not expressed in our conditions. Lack of L-rhamnose, the putative RhaR activating signal molecule, might explain the observed results. For several pectinolytic genes, the regulation of expression on SBP was shown to be dependent on combined control by two or even three TFs. Our results suggest that there might be an antagonistic effect between GaaR and XlnR, because deletion of *gaaR* seems to increase expression of *xlnR* and the main XlnR-dependent targets. The expression of several genes encoding xylan and cellulose-active enzymes was especially highly up-regulated in the triple mutant Δ*gaaR*Δ*araR*Δ*rhaR* (fold changes range 300–500x; Supplementary datasets S9 and S10). This indicates that in the complex TF network that is regulating plant polysaccharide degradation in *A*. *niger*, even apparently not related systems are able to affect each other.

## Methods

### Strains, media and growth conditions

All *A*. *niger* strains used in this study were deposited at the culture collection of the Westerdijk Fungal Biodiversity Institute (previously named CBS-KNAW) under accession numbers indicated in Table 1. Strains were grown at 30 °C in Minimal Medium (MM) or Complete Medium (CM) with appropriate carbon source added^38^. Media of auxotrophic strains were supplemented with 1.22 g L^−1^ uridine. All liquid cultures were incubated in the rotary shaker at 250 rpm. Before protoplasting, the strains were pre-grown for 16 h in Transformation Medium (TM) composed of MM with 5 g L^−1^ yeast extract, 2 g L^−1^ casamino acids and 2% (w/v) glucose. Positive transformants were selected on MM 1.2% (w/v) agar plates with 0.95 M sucrose and 50 mg L^−1^ phleomycin (InvivoGen, Toulouse, France) or 100 mg L^−1^ hygromycin B (InvivoGen, Toulouse, France). To prevent background growth, hygromycin plates were supplemented with 0.5 g L^−1^ caffeine. Phenotypical analysis of the deletion strains were performed in duplicates with MM plates containing 1.5% (w/v) agarose and 25 mM D-glucose, 25 mM L-arabinose, 25 mM D-galacturonic acid, 25 mM L-rhamnose, 1% (w/v) polygalacturonic acid, 1% (w/v) AP (washed and not-washed), 1% (w/v) SBP (washed and not-washed). The pH was adjusted to 6 when necessary. For preparation of the washed pectins, appropriate amount of AP and SBP was mixed with cold water for 5 min, centrifuged and the supernatant containing free monomeric sugars was removed. The remaining pectin pellets were added to MM with 1.5% agarose and autoclaved. Plates were inoculated with 1000 spores in 2 µl of ACES buffer and grown for 6 days. In transfer experiments, freshly harvested spores were pre-grown for 16 h in CM with 2% (w/v) D-fructose. The mycelium was then harvested by filtration over sterile cheesecloth, washed with MM and ~2.5 g (wet weight) was transferred to 50 mL MM with 1% (w/v) sugar beet pectin. After 2 and 8 h of incubation, the mycelium was harvested by vacuum filtration, dried between two sheets of paper and frozen in liquid nitrogen. The culture filtrate was harvested, centrifuged and frozen. All samples were stored in −20°C until being processed.

### Construction of gene deletion strains

Standard molecular biology methods were used for DNA manipulations^39^ unless stated otherwise. The deletion cassettes (containing 5′ and 3′ flanking regions and the selection marker) were constructed using fusion-PCR^40^. For the flanking regions, The ~1000 bp long upstream and downstream DNA fragments of the gene to be deleted were PCR amplified using primers listed in Supplementary dataset S11 and N402 gDNA as a template. For the selection markers, the *Escherichia coli* hygromycin B (*hph*) resistance gene and the *Streptococcus hindustanus* phleomycin (*phle*) resistance gene were amplified from pAN7–1 and pAN8-1 vectors, respectively^41,42^. Correct assembly of the deletion cassettes was tested by restriction enzyme digestion (data not shown).

*A*. *niger* protoplasting and transformation protocol was kindly provided by prof. A. Tsang from Concordia University and is derived from a previously described method^43^ and slightly modified based on^44^. Young mycelia from overnight culture were harvested by vacuum filtration, washed with 0.6 M MgSO_4_ and dried between two sheets of paper. The mycelium was then dissolved in PS buffer (0.2 M sodium phosphate buffer, 0.8 M L-sorbitol, pH6) containing VinoTaste® Pro lysing enzyme (0.5 g enzyme/g of mycelia) and incubated in a rotary shaker at 100 rpm. When protoplasts were abundantly present, the mixture was filtrated through glass wool and undigested mycelia debris were removed. The protoplasts were collected by centrifugation (10 min, 1811 × *g*, 4°C), washed twice with ice-cold SC solution (182.2 g L^−1^ sorbitol, 7.35 g L^−1^ CaCl_2_ * 2H_2_O) and resuspended in SC to a final concentration of 2 * 10^7^ protoplast/mL. For transformation, 200 µL of fresh protoplast suspension, 20 µL of 0.4 M ATA (AurinTricarboxylic Acid ammonium salt), 100 µL of 20% PEG-4000 were mixed with 5 µg of deletion cassette DNA and incubated for 10 min. After addition of 1.5 mL 60% PEG-4000 the mixture was incubated for 20 min. Next, 5 mL 1.2 M sorbitol was added and incubated another 10 min. Transformed protoplasts were collected by centrifugation (10 min, 3220 × *g*), resuspended in 1 mL 1.2 M sorbitol and spread evenly over two selective plates using a cell spreader. Growing colonies were observed after 4 days. Putative deletion strains were purified by two consecutive single colony streaks. gDNA of two independent transformants per strain was isolated using standard phenol/chloroform extraction. Homologous integrations of the deletion cassette were verified by Southern blotting (data not shown). The probes for Southern blotting were DIG-labelled using flanking primers indicated in Supplementary dataset S11, the PCR DIG Probe Synthesis kit (Roche Applied Science) and N402 gDNA as a template.

### Saccharification and identification of enzyme products

Culture filtrates of the reference strain and regulatory mutants grown on SBP for 8 h were used to evaluate degradation efficiency of the strains. Saccharification reactions were assembled in a microtiter plate, three technical replicates and two biological replicates were analyzed. Each reaction contained 20 µL of culture filtrate mixed with 3% (w/v) SBP in 50 mM sodium citrate (pH5) in a final volume of 250 µL. The reactions were incubated for 6 h, with an agitation speed of 400 rpm at 30°C and stopped by heat inactivation of enzymes for 15 min at 95°C. The plate was then centrifuged (10 min, 3000 × *g*), the supernatant was diluted 5-fold in water and analyzed. The enzyme products were identified by High Performance Liquid Chromatography (HPLC) as described previously^45^. Statistical significance was tested using Student’s *t*-test.

### RNA extraction and gene expression analysis

The transcriptomes of the reference strain, single, double and triple mutants induced on SBP for 2 and 8 h were analyzed using RNA-seq. Transfer experiments and subsequent RNA-sequencing were performed in duplicates. RNA was extracted from grinded mycelia using TRIzol® reagent (Invitrogen, Breda, the Netherlands) and purified with NucleoSpin® RNA II Clean-up kit (Macherey-Nagel) with rDNase treatment. The RNA quantity and quality was checked with a RNA6000 Nano Assay using the Agilent 2100 Bioanalyser (Agilent Technologies, Santa Clara, CA, USA). RNA samples were single-end sequenced using Illumina HiSeq^TM^ 2000 platform (http://illumina.com). Purification of mRNA, synthesis of cDNA library and sequencing were conducted at the BGI Tech Solutions Co., Ltd. (Hong Kong, China). Raw reads were produced from the original image data by base calling. On average, ~13 million reads of 51 bp per sample were obtained. After data filtering, the adaptor sequences, highly ‘N’ containing reads (>10% of unknown bases) and low quality reads (more than 50% bases with quality value of <5%) were removed. After data filtering, on average, ~99.3% clean reads remained. Clean reads were then mapped to the genome of Aspergillus niger NRRL3 (http://genome.jgi.doe.gov/Aspni_NRRL3_1) using Bowtie2^46^ and HISAT^47^. On average, 72.8% total mapped reads to the genome was achieved. The gene expression level was measured as Fragments Per Kilobase of transcript per Million mapped reads or FPKM^48^. The multi-mapped reads were removed. For lowly expressed genes, the reads alignment was manually checked using NRRL3 gene models and an Integrative Genomics Viewer (IGV) software^49^. Transcript quantification and differential expression were conducted using the software packages RSEM^50^ and DESeq. 2^51^. A cut-off of fold change > 1.5 between the mutants and the reference strain and adjusted *P*-value ≤ 0.05 were used to identify differentially expressed genes. The heat maps were made by the “gplots” package of R software, with the default parameters: “Complete-linkage clustering method and Euclidean distance”. The RNA-seq data generated in this study has been deposited at the Gene Expression Omnibus (GEO) database with accession number: GSE97974.

### Transcription factor binding motif discovery

RhaR-dependent genes, down-regulated in Δ*rhaR* when compared to the reference strain on L-rhamnose, were selected based on the RNA-seq data generated in our group (Khosravi *et al*., unpublished results). Promoters of 34 putative RhaR targets were extracted form *A*. *niger* NRRL3 v1.0 genome available online at http://genome.jgi.doe.gov/Aspni_NRRL3_1/Aspni_NRRL3_1.home.html. The motif identification was performed with the MEME^37^ software on the set of 34 putative RhaR-binding sequences. The motif width was set from 6 to 10 bases and specified the occurrences to zero or to one per sequence for the MEME search parameters. Only motifs with *E*-value < 1E-02 were used for further analysis. Then, the motif and position-specific probability matrix was generated by MEME to rescan all the promoter sequences of *A*. *niger* NRRL3 v1.0 genome with the FIMO^52^ program. The potential RhaR binding sites were selected with *P*-value < 1E-04.

For motif conservation analysis, the orthologous proteins of 28 putative RhaR-dependent targets were identified by protein sequence similarity search (blastp) against the genome of *A*. *aculeatus* ATCC 16872, *A*. *fumigatus* Af293, *A*. *nidulans* FGSC A4 and *A*. *oryzae* RIB40 available online at JGI MycoCosm (http://genome.jgi.doe.gov/programs/fungi/index.jsf) and AspGD (http://www.aspergillusgenome.org). The orthologous proteins were bi-directional best hits (BBHs) and had minimum 50% sequence identity.

## Electronic supplementary material

Supplementary Dataset 1-12
